# Mechanisms of cell damage due to mechanical impact: an in vitro investigation

**DOI:** 10.1038/s41598-020-68655-2

**Published:** 2020-07-20

**Authors:** Wonmo Kang, Michael C. Robitaille, Marriner Merrill, Kirubel Teferra, Chunghwan Kim, Marc P. Raphael

**Affiliations:** 1 Arizona State University, Tempe, AZ 85287 USA; 2 US Naval Research Laboratory, Washington, DC 20375 USA

**Keywords:** Biomedical engineering, Biological physics

## Abstract

The dynamic response of cells when subjected to mechanical impact has become increasingly relevant for accurate assessment of potential blunt injuries and elucidating underlying injury mechanisms. When exposed to mechanical impact, a biological system such as the human skin, brain, or liver is rapidly accelerated, which could result in blunt injuries. For this reason, an acceleration of greater than > 150 g is the most commonly used criteria for head injury. To understand the main mechanism(s) of blunt injury under such extreme dynamic threats, we have developed an innovative experimental method that applies a well-characterized and -controlled mechanical impact to live cells cultured in a custom-built in vitro setup compatible with live cell microscopy. Our studies using fibroblast cells as a model indicate that input acceleration (${a}_{in}$) alone, even when it is much greater than the typical injury criteria, e.g., ${a}_{in}>1{,}000$ g, does not result in cell damage. On the contrary, we have observed a material-dependent critical pressure value above which a sudden decrease in cell population and cell membrane damage have been observed. We have unambiguously shown that (1) this critical pressure is associated with the onset of cavitation bubbles in a cell culture chamber and (2) the dynamics of cavitation bubbles in the chamber induces localized compressive/tensile pressure cycles, with an amplitude that is considerably greater than the acceleration-induced pressure, to cells. More importantly, the rate of pressure change with time for cavitation-induced pressure is significantly faster (more than ten times) than acceleration-induced pressure. Our in vitro study on the dynamic response of biological systems due to mechanical impact is a crucial step towards understanding potential mechanism(s) of blunt injury and implementing novel therapeutic strategies post-trauma.

## Introduction

Trauma is one of the leading causes of death in the world^1^ and has garnered much interest in recent years due to the realization of the breadth of impact-related pathologies that vary in severity, level of ability for accurate diagnosis, and time scales over which the effects are observed. For instance, blunt liver trauma is often associated with a single severe traumatic incident and might involve immediate surgical intervention once identified^2^. However, traumatic brain injuries (TBI) associated with active military personnel often have a singular traumatic event, yet can manifest as a combination of physical, cognitive or emotional ailments over time that can be misdiagnosed as post-traumatic stress syndrome^3^. Furthermore, the recently discovered prevalence of chronic traumatic encephalopathy (CTE) in contact sports athletes is associated with compounding effects of less-severe, yet still traumatic events over time that can only be accurately diagnosed posthumously^4^.

Many resources and efforts have been utilized over the years to better understand the pathophysiology of blunt impact injuries. Typically, animal models that utilize injury mechanisms to the organ of interest in conjunction with end-point assays, such as immunohistochemistry, have been a common platform to identify injury related biomarkers or to assess cell/tissue damage^5^. However, the mechanisms underlying post-trauma cellular damage or death remain poorly understood^6^, likely in part due to the lack of standard testing procedures and the complexity of interpreting animal models^7^. This has prompted research efforts to focus on simpler in-vitro platforms, allowing researchers to investigate potential underlying injury mechanisms at the cellular or molecular level^8^. However, in many of these in-vitro platforms, it has proven technically challenging to accurately apply injury-relevant pressure forces to the cell population in question in tightly controlled environments^9^.

The human body is exposed to considerable linear acceleration in the range of 80–250 g during mechanical collisions, e.g., football and boxing matches^10,11^ or military operations^12,13^. Such rapid acceleration of the human body results in various traumatic injuries including blunt liver trauma^2,14^ or traumatic brain injury^10–12,15^. It is important to note that acceleration alone is the most commonly used criteria for assessment of blunt injury.

Dynamic cavitation in the brain is increasingly considered a potential damage mechanism for traumatic brain injury^16–20^. In this regard, one notable advance is in the characterization of cavitation properties for soft biomaterials under an impulsive force^21–23^. In these studies, the critical acceleration that corresponds to the onset of the bubble formation and bursts has been experimentally and theoretically characterized utilizing biologically relevant soft biomaterials including collagen, agarose, and gelatin. These studies have established a range of mechanical acceleration for potential cavitation-induced brain injury. Further, these studies have shown that the acceleration-induced local pressure strongly depends on the sample size, and therefore, in addition to acceleration, the size of biological system experiencing trauma, e.g., head, should be considered for accurate assessment of potential injuries.

Despite the recent progress on the dynamic response of tissue simulant, experimental studies on the spatio-temporal response of live cells under well-characterized acceleration-induced pressure are very limited in the literature largely due to the lack of such experimental capability. Some of the key experimental challenges include: (1) maintenance of consistent in vitro conditions during the experiments and observation periods; (2) a capability to probe cells in time, in particular before and after impact; (3) a high throughput approach for analyses of multiplexed cell populations. The combination of these capabilities are necessary for revealing cellular behavior associated with injury mechanisms and ensures statistical significance considering the heterogeneous nature of cell response. For example, understanding and characterizing the cell-line specific senescence for in-vitro platforms is of paramount importance to accurately probe underlying mechanisms and ensure reproducible results.

Here we investigate damage mechanisms of live cells to address our key question of “what are the key injury mechanisms, e.g., acceleration or pressure, and injury criteria associated with mechanical impact?” Towards this fundamental question, we have developed and utilized a new experimental approach for the application of well-controlled impact to live cell populations in vitro. First, we describe a new drop-tower-based experimental setup designed to apply a wide range of blunt injury mimicking accelerations to live cell populations, while also optically monitoring the in vitro cell culture. Then we theoretically consider pressure in a cell culture chamber during impact to establish an acceleration-pressure relation during impact. Utilizing the new experimental capability, we experimentally evaluate damage to cells, by using fibroblast cells as a model, during impact. Finally, we quantify the acceleration- and cavitation-induced pressure during impact and show the latter is the critical injury mechanism to cells due to the dynamic nature of the cavitation.

## Experiment setup

Here we present a new technique for studying cell injury mechanisms by applying biologically relevant mechanical impact to in vitro cell culture. This new approach is for maintaining consistent in vitro conditions during experiments, accommodating multiple cell populations, and monitoring each population in real-time while the impact-induced accelerations which mimic blunt injury are quantified with regards to amplitude and time scale. These multiplexed, environmental control capabilities are critical for studying the relationships between mechanical impact and cell injury due to the complex nature of interpreting input–output relationships in multivariate biological systems.

### In vitro setup for blunt injury mechanisms

**Figure 1:**
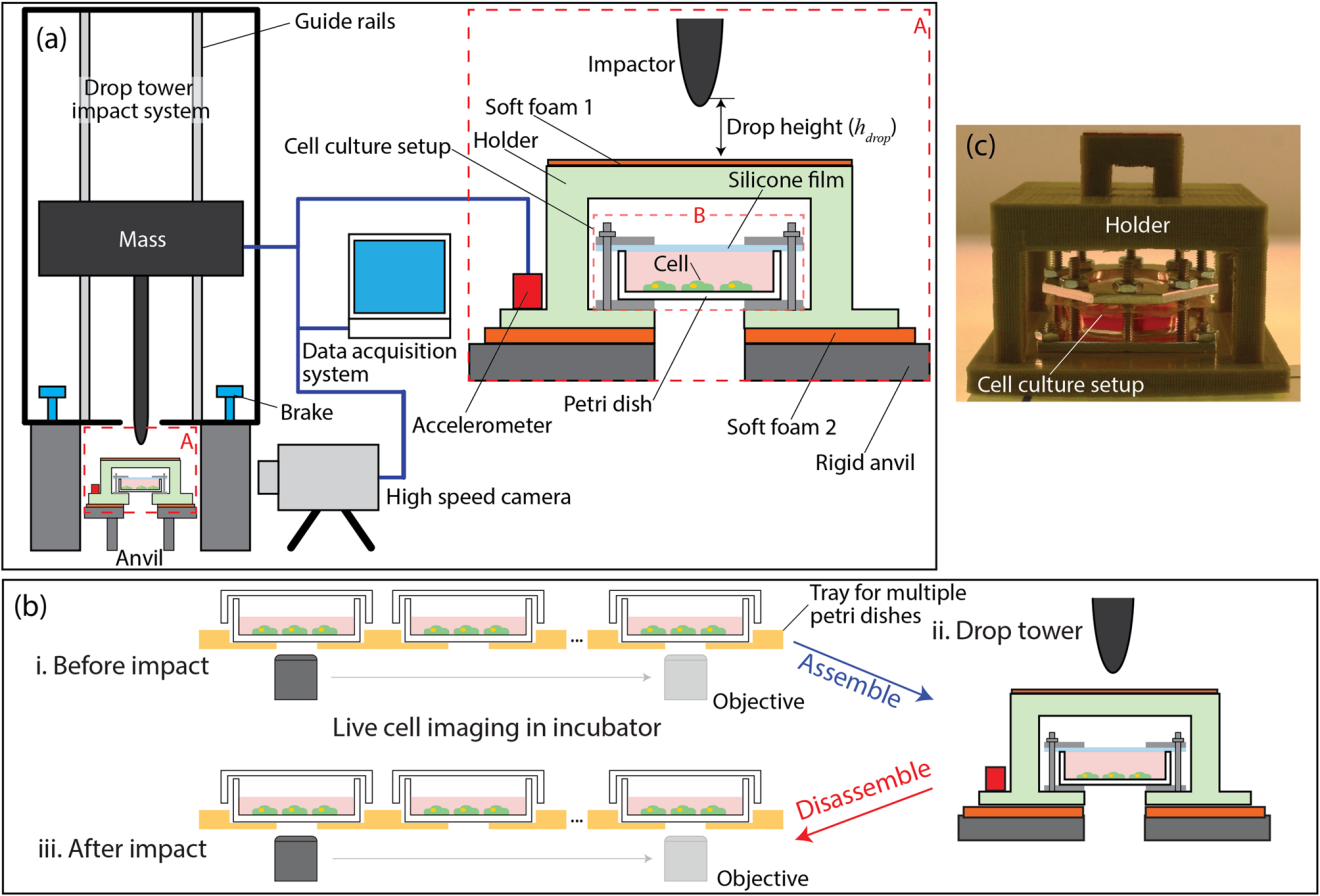
(**a**) Schematic of the drop-tower-based integrated system for characterizing the biological response of live cells due to rapid acceleration associated with mechanical impact. The inset (zoom-in view of *Area A*) on the right shows the structures of the cell culture setup (highlighted by *Area B*) and the holder with electrical connections for data acquisition. (**b**) Shows the experimental protocol to study acceleration-induced cell damage utilizing both the live cell microscopy and drop tower based instrument. (**c**) An image of an assembled holder with a cell culture setup for a drop tower experiment. (**b-i**) Prepare multiple cell culture petri dishes and monitor them using live cell imaging capability in the incubator. All the prepared cell culture dishes are placed on a tray in a cell culture incubator and a motorized optical objective is used to take live cell images for all dishes at specific time intervals (see the arrows). When cell populations reach a specific target stage, e.g., 35–40% confluency in the cell growth curve, (**b-ii**) each petri dish is assembled with a holder for drop tower experiments (**c** shows a petri dish assembled with a cell culture setup, see Figure S1 for more details). (**b-iii**) After a drop tower experiment, a petri dish is disassembled from the cell culture setup and placed in a cell culture incubator for continuing live cell imaging.

Acceleration for typical blunt injuries, as discussed above, is in the range of 80–250 g within 0.1–10 ms. Considering these specifications, it is important to control collisions so that they closely represent biologically relevant blunt injury events during in vitro studies. In this regard, we have developed and utilized a new in vitro setup that can be integrated with a drop-tower-based system as well as a multiplexed, live cell-imaging instrument, Fig. 1a.

The system consists of a conventional drop tower impact system, two high speed cameras, a cell culture setup, accelerometers, and a data-acquisition system. For applying impact on the cell culture setup, a weight is vertically lifted to a specific height and then released. Upon the release, the weight impacts a holder with an integrated cell culture setup causing the cells and cell culture media to rapidly accelerate in the similar way biological systems would accelerate, e.g., inside the abdomen or skull. During impact, velocity, acceleration, and impact force are quantified using various sensors, while optically monitoring the cell culture setup utilizing high-speed cameras. Before and after an impact experiment, the cell culture petri dish is kept in incubator (Fig. 1b-i and -ii) for various analyses via live cell microscopy.

The cell culture setup (*Area B* in Fig. 1a) consists of a commercially available 35-mm cell culture petri dish, aluminum plates, and a transparent silicone film. Each petri dish is assembled/disassembled with the cell culture setup by standard bolts and nuts with other components. Two major technical challenges for any in-vitro experimental platform are environmental control and cell heterogeneity. First, cells are sensitive to changes in their environment and, as a result, minimizing and eliminating unwanted perturbations to cellular environments during in vitro studies is imperative for accurate interpretation and reproducibility of results. Second, both individual and collective cellular behavior is heterogeneous in nature, potentially confounding the interpretation of cellular response associated with the injury mechanism. The cell culture setup is designed to address these challenges by integrating with a multiplexed, live cell-imaging instrument as schematically shown in Fig. 1b-i and -iii (see Figure S1 in the Supplementary document for structural details of the cell culture setup).

It is important to note that the holder is rigid enough to sustain impacts while preventing direct impact to the cell culture setup. The two soft foam layers (*Soft foam 1* and *2* in Fig. 1a) are used at the top and bottom of the holder, critical to achieve desired impact in terms of both amplitude and time scale of acceleration. The top layer (*Soft foam 1*) is thinner (1 mm) and stiffer (4 MPa) to prevent direct solid–solid surface impact, which would generate shock waves, while effectively accelerating the holder (see Figure S2 in the supplement document). The bottom layer (*Soft foam 2*) is thicker (12 mm) and softer (0.4 MPa) so that the holder can vertically move against the small stiffness of the layer. These parameters are specifically chosen to simulate common blunt injury scenarios, i.e., > 150 g and < 1 ms. This experimental setup is generalizable so that the system parameters, e.g., the soft foams, can be tuned to mimic characteristics of different blunt injury cases as reported in great detail^21^.

Our new approach allows continuous optical observation of multiple cell culture setups (currently up to 12 separate culture setups) without the need to take cells out from an incubator for observation. Additionally, the multiplexing capability of multiple cell populations in parallel offers a unique dynamic window into how cells respond to mechanical input by directly comparing the same population of cells before and after impact in real-time. These attractive capabilities are essential for probing the injury mechanisms of cells while minimizing batch-to-batch and/or population-to-population variations in cell response due to heterogeneities.

### Theoretical analysis on acceleration-induced pressure

Acceleration-induced pressure profiles during impact are a primary mechanism for blunt injuries. Therefore, it is necessary to characterize the pressure-acceleration relationship in the cell culture setup to understand the intimate connection between cell damage and mechanical impact.

**Figure 2:**
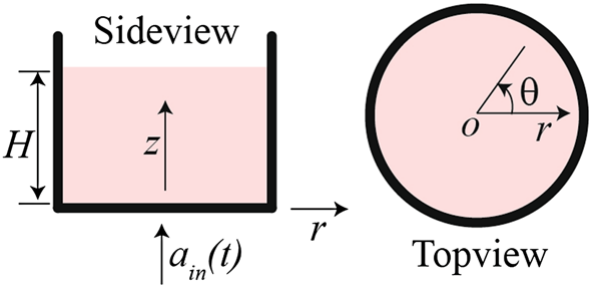
Transverse and cross sectional views of the cell culture setup denoted by a cylindrical polar coordinate system.

Consider the transverse and cross sectional views in Fig. 2 that presents the cell culture setup with a focus on the time-dependent pressure profile in the cell culture media during impact where $d$ and $H$ are the diameter and height of the cell culture chamber, respectively. $r$, $\theta$, and $z$ are the radial, angular, and vertical coordinates with respect to the origin at the center of the bottom surface of the cell culture chamber. Upon mechanical impact on the holder, the cell culture setup is rapidly accelerated by ${a}_{in}$ that results in acceleration-induced pressure (${p}_{a}$) in the cell culture media where ${a}_{in}$ and ${p}_{a}$ are functions of time ($t$).

In the following analysis, we assume that the cell culture media is incompressible Newtonian fluid. Due to the axial symmetry of the cylinder shaped cell culture chamber about $z$-axis, the pressure gradient (${p}_{in}$) in the cell culture media depends on two coordinates, $r$ and $z$, only, and as a result all terms with a partial derivative of $\theta$ are zero, i.e., $\partial /\partial \theta =0$ (more details are discussed in the supplementary document). We also assume no slip conditions along the solid–liquid interfaces at $(r, z)=(d/2, z)$ and $=(r, 0)$. Note that all external forces acting on the cell culture media during impact are associated with the no slip boundary conditions at the solid–liquid interfaces. Because all external forces are in $z$-direction, we assume the velocity of the cell media in $r$- and $\theta$-directions is zero. Based on the experimental observations provided in the supplement, we have shown that our assumptions on the velocity are reasonable between the time points from the onset of impact to maximum amplitude of input acceleration.

Using the assumptions above, the governing equation of motion for incompressible Newtonian fluid, i.e., the Navier–Stokes equations^24^ with constant density of cell culture media (i.e., $\rho (r,\theta ,z,t)=constant$), in the cylindrical polar coordinates can be simplified as below

1 $$\rho \left({a}_{in}(t)-g\right)=-\frac{\partial {p}_{a}}{\partial z}$$

where $g$ is gravity (see Eqs. S1–S3 for details in the supplement). By solving Eq. 1 using the free surface boundary (${p}_{a}(z=H)=0$), the acceleration-induced pressure in the cell culture media can be written as

2 $${p}_{a}\left(z, t\right)=-\rho \left(H-z\right)\left({a}_{in}\left(t\right)-g\right)$$

Note that the pressure in the cell culture media is linearly proportional to depth of cell culture media ($H-z$) and input acceleration (${a}_{in}$). The most important location is the cell culture surface at z = 0 in which the pressure applied to cells is ${p}_{a}\left(0, t\right)\approx -\rho H{a}_{in}(t)$ for ${a}_{in}\gg g$.

## The key injury mechanism

Acceleration is the most commonly used criteria for blunt injury mainly due to ease of quantification, e.g., when compared to direct pressure measurements inside the human body. In addition to the easier measurement, it can be argued that acceleration could be a good representation of time-dependent pressure gradient in biological systems because acceleration and acceleration-induced pressure are intrinsically coupled during blunt impact, as suggested by Eq. 2. However, the acceleration-based injury criteria have greatly overlooked the fact that acceleration-induced pressure also depends on the characteristic length of the biological systems, i.e., the height of cell culture media ($H$) in Eq. 2. In addition, the recent experimental work utilizing collagen as tissue simulant has shown a non-linear acceleration-induced pressure for soft biomaterials^23^, e.g., tissue, indicating that liquid based experiments may even underestimate the sensitivity of cells to $H$.

To explore possible effects of size on blunt injury mechanisms, we utilize a cell culture chamber ($H=10$ mm) and characterize changes in cell response of fibroblast associated with mechanical impact. Our focus is on quantifying the critical input acceleration for cell damage by monitoring cell confluency and cell membrane perturbations. Then we consider the possible underlying mechanism(s) that govern the correlation between the critical input acceleration and the onset of the detectable damage to cell populations.

### Multiplexed in vitro cell cultures

**Figure 3:**
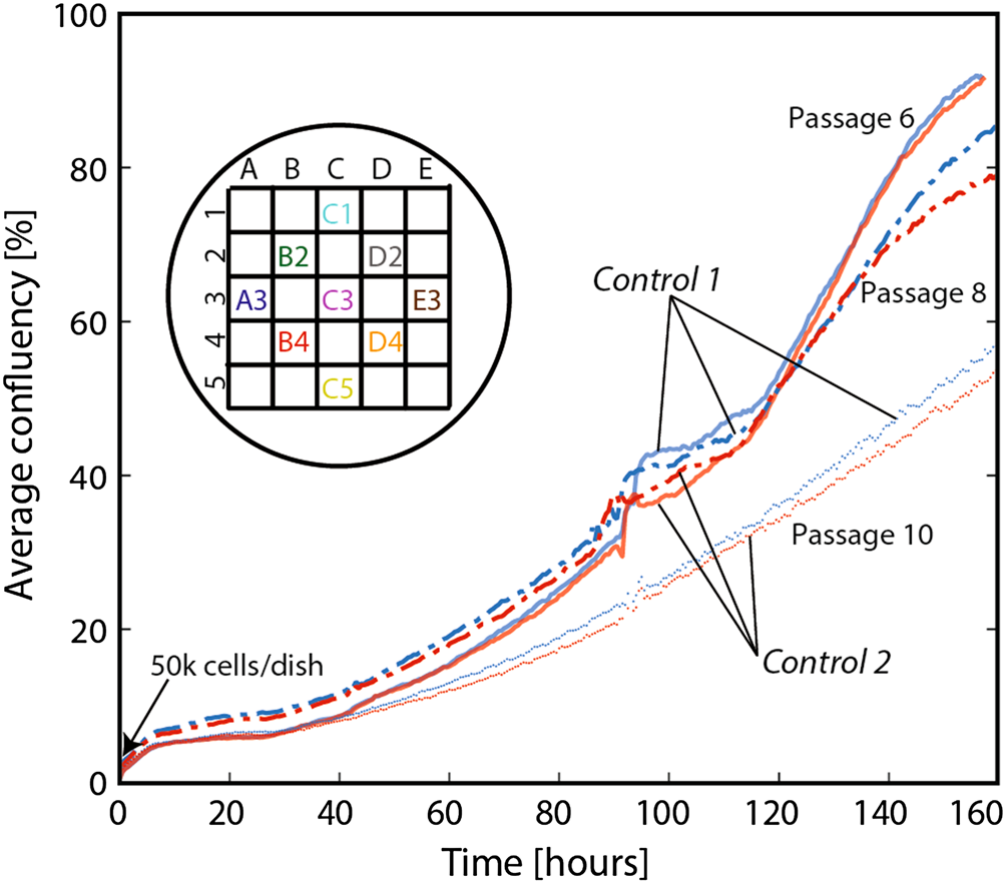
The average confluence of two *controls* without being exposed to mechanical impact for Hs27 cells. The average confluency was evaluated from the nine areas on each 35 mm petri dish (A3 to E3). *Control 1*: Additional cell media was added and immediately returned to an incubator (i.e., stayed in an incubator during entire experiments). *Control 2*: After adding additional cell media, *Control 2* underwent the exact conditions of the *non-control dishes*, i.e., assembled and stored in a temperature-controlled box for ~ 10 min, except for application of impact.

For the experimental study of cell damage due to mechanical impact, we first prepared multiple cell culture petri dishes and monitored them using live cell imaging capability in an incubator, Fig. 1b-i. After the cells cultured on an individual petri dish reached a specific target stage, e.g., 35–40% confluency in the cell growth curve, additional cell culture media was added to the dish before assembling with a holder for the drop tower experiments, Fig. 1b-ii. Note that two dishes were always dedicated for control experiments (specific conditions for the *controls* are discussed below), while *other dishes* were for drop tower experiments. To quantify cell population, we continuously monitored the local confluency at each area from A3 to E3 (see the inset in Fig. 3) as well as the average confluency of each dish (the average over the nine areas) during cell culture concurrently for both the *controls* and *other dishes*. The confluency is the ratio of the area covered by cells to the total area, evaluated based on image analysis of cells (see “Method” section for more details).

It is important that extra cell culture media was added to the maximum capacity of a petri dish (~ 10 mm in height) only during the assembly of the drop tower holder as shown in Fig. 1b. This step ensured (1) 3–5 mm deep cell culture media was maintained during regular cell culture and (2) excessive air was removed from the cell culture setup during drop tower experiments. First, the shallow cell culture media is important for CO_2_ exchange that ensures media pH stability during culture^25^. Second, we have found that a trapped air pocket between cell culture media and a silicone film (Fig. 1a, *Area B*) during the assembly of the cell culture setup could result in unwanted cell damage during impact. As an example, we have observed (see Supplementary Movie 1) that the trapped air was inserted into the cell culture media during violent impact. In this case, cells could be damaged when directly exposed to the air, which is not a biologically relevant mechanism.

Unless stated otherwise, the same volume of cell culture media, like *the other dishes*, was added to the negative *control dishes* for maintaining consistent in vitro conditions during experiments. After adding media, one dish (*Control 1*) was immediately returned to an incubator without being assembled with the cell culture setup while a second (*Control 2*) underwent the exact conditions of the *non-control dishes*, i.e., assembled and stored in a temperature-controlled box for ~ 10 min, except for application of impact. *Control 1* and *2* were designed to quantify possible cell damage associated with the assembly procedure of the cell culture setup.

Figure 3 shows the average confluency of fibroblast cells for *Control 1* and *2* prepared from *passages 6, 8, and 10*, respectively. The confluency curves of the two controls are very comparable to each other at all three passages. This study indicates that the new in vitro cell culture setup minimizes unwanted perturbations to cell cultures for ~ 1 week.

There are a number of interesting observations by making multiplexed, real time measurements of the growth as opposed to the more typical end-point assay approach to in vitro studies. For instance, in Fig. 3 the growth rate becomes noticeably slower for *passage 10* compared to the previous passages, which is important for identifying passage-dependent responses. In addition, local confluency curves of each region of interest can be significantly different from the average values (see Figure S5). The observed, intrinsic temporal and spatial cell heterogeneity could mask or mislead studies on injury mechanisms. As will be shown below, making conclusions based on the data from a localized small area could be misleading because each area even on the same petri dish could have significantly different cell population/behavior/growth compared to the average cell behavior. The examples above highlight the attractive features of our approach including (1) characterization of the multiple, identical cell culture populations in real time to a variety of impact accelerations and pressure gradients and (2) a capability to analyze a sufficiently large cell population for statistically significant results.

### The critical blunt injury conditions

**Figure 4:**
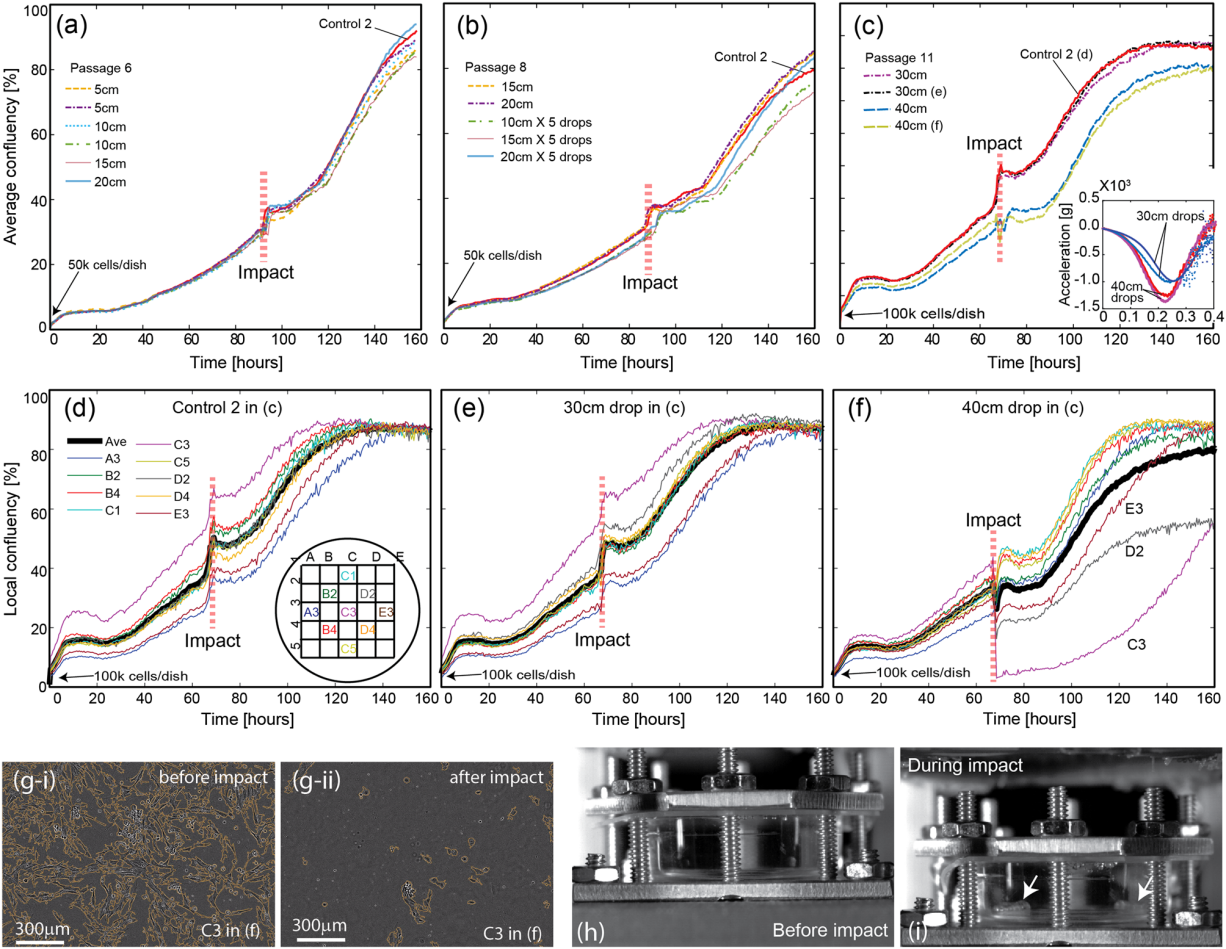
Characterization of the critical mechanical impact for cell injury using Hs27 cells using an average confluency metric (**a**–**c**) versus a local area confluency metric (**d**–**f**), monitored over 160 h. The average confluency curves (**a**) 5 cm-, 10 cm-, 15 cm-, and 20 cm-single-drops, (**b**) 15 cm-/20 cm-single-drops as well as 10 cm-, 15 cm-, and 20 cm-five-drops, (**c**) 30 cm- and 40 cm-single-drops on the cell cultures. Passage 6, 8, and 11 were used for (**a**), (**b**), and (**c**), respectively. The acceleration measured during 30 cm- and 40 cm-drops are shown in the inset of (**c**). The local confluency curves for *Control 2*, 30 cm-drop, and 40 cm-drop from (**c**) are shown in (**d**–**f**), respectively. (**g-i**) and (**-ii**) are live cell images corresponsding to area C3 in (**f**) before and after the impact, respectivly (also see the Supplementary Movie 2). (**h**) and (**i**) are high speed camera images of the cell culture setup during 40 cm-drop. Cavitation bubbles are observed on the petri dish (see the arrows in (**i**)).

Here we quantified the critical blunt injury conditions that resulted in cell damage to fibroblast cells. For this study, several cell cultures prepared from passages 6, 8, and 11 (the same cell culture used in Fig. 3) were subjected to a range of drop tower heights from 5 to 40 cm during the middle (35–40%) of their logarithmic growth phase (see “Impact” in Fig. 4a–f). Note that Figs. 3 and 4 are based on more than 20 independent petri dishes (e.g., each curve in Figs. 3 and 4a–c is obtained from independent individual petri dishes) and at least two petri dishes were cultured and tested under the same conditions. The cell cultures that were exposed to less than 40 cm drop, both single/multiple impact conditions, show the average as well as local confluency curves that are very similar to the corresponding *Control 2* results (see Fig. 4a,b,d–f for the average and local confluency curves, respectively). On the contrary, the average confluency after the 40 cm-drop experiment decreased from 48 to 35% (Fig. 4c). Furthermore, the local confluency curves (Fig. 4d–f) indicated that the cell damage after 40 cm-drop was not homogeneous over the entire petri dish as significant cell population losses were localized to C3, D2, and E3 (Fig. 4f). As an example, the local confluency in C3 significantly dropped from ~ 40 to ~ 3%. This dramatic change is also shown in g-i and -ii, live cell images before and after 40 cm-drop, respectively. Note that the orange lines in g-i and -ii highlight the contours of cells.

Based on the experimental data summarized in Fig. 4, we have concluded that the critical drop height associated with the noticeable loss of cell population was > 30 cm. Interestingly, the maximum amplitude of input acceleration for 30 cm-drop was 990–1,000 g (see the inset in Fig. 4c), significantly larger than the commonly used criteria (150 g) for blunt head injury. Equation 2 could provide a feasible explanation for this. The maximum acceleration-induced pressure is linearly proportional to the size of biological system and, as a result, the critical input acceleration associated with cell damage would significantly increase with decreasing size of biological systems.

Another unexpected observation was a sudden increase in localized cell death for 40 cm-drops, rather than globally and incrementally increasing cell death, where the corresponding amplitude of acceleration was 1,280–1,370 g (see the inset in c). To identify the specific damage mechanism that seems to be activated for > 1,000 g, high-speed camera movies were utilized (see Fig. 4h,i and the Supplementary Movie 3). The high speed images in h-i show that cavitation bubbles, likely responsible for localized cell damages considering violent nature of cavitation dynamics, formed at the bottom of a petri dish for 40 cm-drops. We further discuss this cavitation-induced mechanism for cell damage later.

To support the two conclusions above, the detailed confluency curves with the standard error (95% interval) are shown in Figure S5-1 to 6 (see the supplement document). Our emphasis is placed on experimental data presented in Fig. 4c because the figure can be used to determine the critical drop height that triggers statistically relevant decreases in the confluency curves. Note that among Figure S5-1 to 6, statistically relevant cell damage was observed only for the 40 cm drop experiments and was limited to local ROIs, (e.g., see B2 in Figure S5-5 and C3 and D2 in Figure S5-6).

To further highlight the importance of system size, we note that the critical acceleration (~ 300 g) at the onset of cavitation in pure water was recently quantified using a 40 mm-height cuvette. Note that ~ 1,300 g, which triggers cavitation in the 10 mm-height cell culture setup, is about four times larger due to size dependent acceleration-induced pressure (Eq. 2). Note that the incorporation of a pressure measurement probe in the cell culture setup, while desirable, is still very challenging because insertion of such a sensor will likely contaminate cell culture.

**Figure 5:**
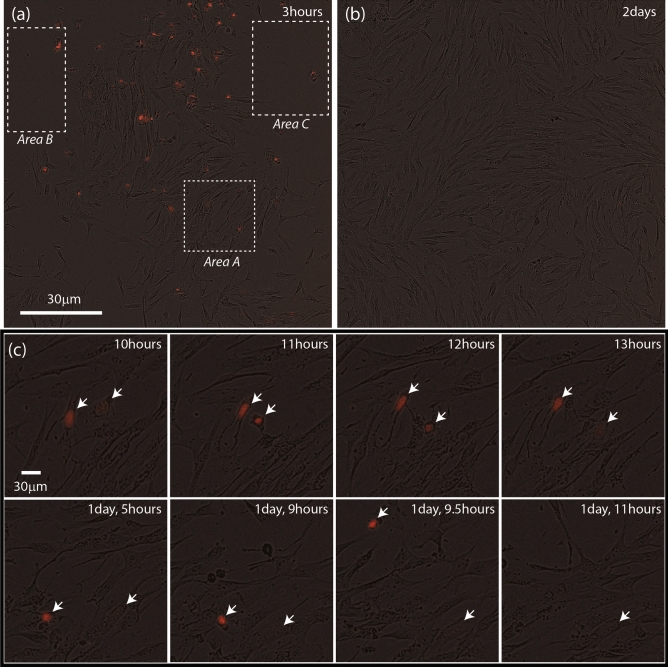
Propidium iodide (PI) fluorescence intensity overlaid with phase imagery highlighting PI of fibroblasts post-impact as a function of time. (**a**,**b**) are 3 h and 2 days, respectively, after the 40 cm-drop impact where *Area B* and *C* highlight localized loss of cell population due to cavitation. (**c**) is time lapse of *Area A* in (**a**).

Because of the importance of cell membrane integrity in cell injury mechanisms, we considered membrane permeabilization associated with pressure gradients. For example, it is well known that mechanical damage to cell membranes could result in irreversible cell death by necrosis^26^ by glutamate release from the damaged cells or off balance of intracellular ion concentrations^27^. To monitor the plasma membrane integrity of individual cell populations in real time after the 40 cm-drop, we implemented a propidium iodide (PI) assay. Cells stained with this dye indicate a compromised plasma membrane because PI is impermeable to healthy cells (see the method section for detailed procedure). Figure 5a shows significantly damaged areas (see *Area B* and *C*, also see the supplementary document for the criteria to determine area damage) on a petri dish. Among the remaining cells, approximately 10% of fibroblasts in this region were stained by PI 3 h after impact. Interestingly, 48 h after impact, the cell population had doubled and no cells stained with PI were observed (Fig. 5b).

To assess the fate of cells showing positive PI staining, we analyzed the relative PI levels in individual cells over time. Figure 5c is the zoom-in view of *Area A* in (a) and shows time lapse imagery of a cluster of cells initially stained with PI (arrows). The morphology and positions of the cells changed over the course of 24 h, indicating that the fibroblasts were motile after impact. One cell exhibited a significantly reduced fluorescence intensity after 3 h, whereas the 2nd cell exhibited a more gradual reduction in fluorescence intensity over the course of 25 h. At 24 h, a new cell stained with a large fluorescence intensity entered the region, suggesting that the reduction in cell PI staining was not due to photobleaching. 48 h after impact, no PI staining was observed in the cell population, indicating that fibroblasts plasma membrane was repaired over time. It is worth noting that cell PI staining was observed only near damaged areas, which indicates that the cavitation-induced membrane damage is localized (Figure S6 in the supplementary document).

So far, we have concluded that acceleration alone does not damage fibroblasts even when the applied acceleration (~ 1,000 g) is significantly greater than the conventional injury criterion, 150 g. In contrast, mechanical impact corresponding to the onset of cavitation bubbles results in sudden cell damage. Because the dynamics of cavitation bubbles is stochastic and localized (see Supplementary Movie 3, 40 cm drop), significant loss of cell population is limited to randomly distributed, localized spots on a 2D cell culture plate in the cell culture chamber. Among the remaining cells in the damaged spots, cell membrane damage has been detected utilizing fluorescent imaging techniques. While these results are consistent with membrane poration, more studies are needed for quantitative conclusions for acceleration-induced cell membrane permeabilization as well as for fundamentally understanding cavitation’s role in cell membrane damage.

## The main injury mechanism: cavitation

Interestingly, acceleration-induced pressure did not result in cell detachment nor change in cell growth curves when ${a}_{in}<{a}_{cr}$ where ${a}_{in}$ is the amplitude of input acceleration and ${a}_{cr}$ is the critical acceleration for cavitation. In this case, the acceleration-induced pressure at the bottom of a petri dish (where cells are growing) during the impact event ($0<t<1$ ms) was expected to be tensile using Eq. 2. In addition, the amplitude of tensile pressure would be less than the critical pressure for cavitation nucleation in pure liquid, 110 kPa^17,21^. On the other hand, the cells that were directly under the influence of cavitation bubbles were significantly damaged.

One possible explanation for this significant difference in cell response before/after impact is that the amplitude of cavitation-induced pressure may be much larger than acceleration-induced pressure. However, a recent study reported that pressure associated with bubble collapse of thermally induced cavitation is about 56 kPa^19^, which is considerably smaller than the critical pressure, using a pressure sensitive film. Another possible mechanism is that cavitation-induced pressure, i.e., a rate of the pressure change in time, is much faster than acceleration-induced pressure and, as a result, viscoelastic response of cells, i.e., cell damage response, at different loading rates could be significantly different^28^.

**Figure 6:**
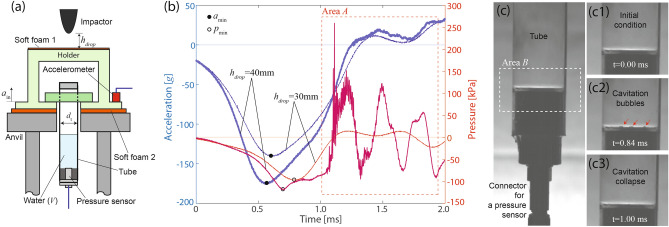
Direct comparison between acceleration (${a}_{in}$) and acceleration-induced pressure (${p}_{m}$) during impact. (**a**) Schematic of an experimental setup for concurrently monitoring acceleration and pressure. (**b**) Experimental data to compare acceleration (vertical axis on the left, blue) with the corresponding pressure (vertical axis on the right, red) in time for pure water (*V* = 6 ml) in a transparent plastic tube. (**c**) High speed camera images of the tube during 40 mm-drop.

To quantitatively consider the two possible damage mechanisms above, we prepared transparent plastic tubes with an inserted pressure sensor for measuring time-varying pressure at the bottom of the tube. Figure 6a,c show a schematic and an optical image of a transparent plastic tube filled with 6 ml pure water where a pressure sensor is inserted at the bottom. This experiment setup allows concurrent measurements of acceleration and pressure at the liquid-plate interface, which mimics the cell culture media and plate interface at the bottom of the cell culture chamber.

The experimental setup was designed to achieve a higher probability of cavitation bubbles nucleation directly on a pressure sensor. First, while cavitation nucleation is stochastic in nature, bubbles would most likely form at the bottom of the tube where the amplitude of acceleration-induced pressure (see Eq. 2) becomes maximum. Second, assuming a cavitation bubble occurs at the bottom plane, we can effectively decrease the expected distance between cavitation bubbles and a fixed-area pressure sensor by decreasing a cross sectional area of a tube. As an example, the probability of having a cavitation bubble directly on a pressure sensor ($d$ ~ 5.5 mm) could increase by more than 8 times by simply decreasing a cross section from $d$ ~ 35 mm (petri dish) to ~ 12 mm (tube) due to an increase in the ratio of sensor to available surface areas where $d$ is diameter.

Figure 6b shows acceleration and acceleration-induced pressure for 30 mm and 40 mm drop experiments utilizing a plastic tube filled with 6 ml pure water ($H\sim 40$ mm). For ${h}_{drop}=30$ mm (also see Supplementary Movie 4), the smooth profile of the acceleration signal and measured pressure was very similar with a small shift in phase likely due to the viscosity of water. The measured amplitudes of acceleration and pressure were ${a}_{min}=-140.2$ *G* and ${p}_{min}=-96.9$ kPa, respectively. In addition, shock waves, i.e., pulse-like signals (see the dotted lines in Figure S2b), were not observed in the pressure measurement. Note that the critical tensile pressure for cavitation nucleation in water is about 110 kPa.

For ${h}_{drop}=40$ mm, qualitative trends within $t<1$ ms were very similar to ${h}_{drop}=30$ mm. As expected, the amplitudes of acceleration and pressure increased to ${a}_{min}=-174.7$ *G* and ${p}_{min}=-118.6$ kPa, respectively, due to the increasing drop height. Although the qualitative acceleration profiles of the both drops did not change much, the corresponding pressure measurement for ${h}_{drop}=40$ mm became significantly different for $t>1$ ms, i.e., rapid tensile/compressive pressure cycles superposed with much higher frequency acceleration signals.

To reveal a main mechanism that corresponds to the sudden changes in pressure, we performed image analysis of high-speed camera movies that were synchronized with acceleration and pressure measurements in time. Figure 6c1–c3 shows the zoom-in view of *Area B* in (c), which is a likely cavitation nucleation site due to the maximum local pressure, at different time points. The cavitation nucleation was first detected around $t=0.64$ ms. The bubbles grew in size until $t=0.84$ ms and then fully collapsed after $t=1.00$ ms (also see Supplement Movie 4), which correlates with the rapid tensile/compressive pressure cycles observed in Fig. 6b. These images strongly indicate that the sudden changes in the pressure were mainly associated with dynamics of cavitation bubbles, in particular, cavitation collapse.

Two important conclusions from the experimental data in Fig. 6 are (1) the amplitude of cavitation-induced pressure is considerably larger than acceleration-induced pressure and (2) a rate of pressure changes for cavitation-induced pressure is significantly faster than acceleration-induced pressure. Upon impact, the amplitude of acceleration-induced pressure gradually increases from 0 at $t=0$ ms to 118.6 kPa at $t=0.70$ ms. On the other hands, cavitation-induced pressure increases from − 72.9 to 259.2 kPa within 0.05 ms where the maximum pressure occurs at $t=1.11$ ms. Note that the corresponding rates of pressure change for acceleration- and cavitation-induced pressure are 169 kPa/ms and 6,642 kPa/ms, respectively. It is worth mentioning that the pressure measurement is an average over the entire sensor and, therefore, the localized pressure must be even higher near the cavitation event.

It is worth discussing that the acceleration-induced pressure ($t<1$ ms) for both ${h}_{drop}=30$ and $=40$ mm strongly suggests that the 1 mm-thick foam at the top of the sample holder effectively eliminates possible propagation of shock waves to the liquid and pressure in the liquid is indeed acceleration-induced. One possible implication of these results is that the effect of shock waves on mild blunt injury is dampened because the human body is protected by soft skin, which would behave as the thin foam layer.

## Conclusion and discussion

Mechanical impact and the resulting acceleration-induced pressure gradient on the human body is one of the main mechanisms for traumatic injury. Fundamental understanding of injury mechanisms associated with mechanical impact is essential toward the development of reliable injury criteria and more accurate prediction of injury. In addition, such knowledge could pave the way for innovative protective equipment designs and effective strategies for post-injury treatment.

Here, we have introduced a novel platform capable of applying well-characterized and reproducible acceleration-induced pressure gradients that is compatible with long-term and environmentally controlled live-cell microscopy. This in vitro platform allows close monitoring of multiple cell populations, down to individual cells, to deduce how impact effects cell viability and mitosis, as well as assays for investigating the molecular mechanisms of cell injury, such as membrane permeabilization due to pressure gradients.

Our drop tower studies using the cell culture setup in vitro reveal that even a seemingly very large acceleration (~ 1,000 g) does not result in apparent cell damage due to the small size (e.g. height) of the cell culture setup (~ 10 mm). This result indeed emphasizes the importance of appropriately considering the effect of biological system size, in addition to acceleration, for accurate assessment of blunt injury. As an example, human head size could be an important factor to predict blunt head injury among different sample populations, e.g., female vs male or child vs adult, for a given acceleration.

In addition, we have shown that sudden cell death is triggered by the onset of cavitation bubbles, which apply localized compressive/tensile pressure cycles to cells, in the culture chamber. We have found the cavitation-induced pressure is much more violent than acceleration-induced pressure due to a very rapid rate of pressure changes in time and large pressure amplitudes relative to when no cavitation is present.

This platform offers a novel window into fundamental mechanisms of cell injury due to impact. The integration with standard cell culture platforms allows future work to address fundamental questions such as: how does impact affect cell adhesion, motility, and mitosis? Indeed, novel migration or adhesion assays conducted on standard cell culture dishes could be incorporated into the in vitro impact tower set up described here. Furthermore, this platform could be expanded to a plethora of cell lines, such as neurons, or in vitro conditions, such as serum free media or altered substrate stiffness.

The focus of the current work is placed on assessing cellular damage at the population level associated with rapid mechanical acceleration and the subsequent onset of cavitation bubbles. Because the current experimental setup does not allow real-time measurements of deformation of individual cells during impact, fundamental mechanistic information on critical parameters required to assess damage at the single cell level is lacking. To elucidate critical parameters for single cell damage, future efforts will integrate the current system with high resolution imaging techniques to quantitatively correlate the critical impact amplitudes, pressures, and cell deformation with a specified damage to individual cells.

## Method

### Cell culture and image analysis

For initial validation of this platform, Hs27 fibroblast cells (ATCC, CRL-1634) cultured in DMEM (ATCC 30-2002) and 10% FBS (ATCC 30-2020) are used for all experiments. Fibroblasts are initially plated in several T25 flasks (Corning), with at least one flask solely used to measure and characterize cell confluence as a function of passage. A separate flask is harvested during its logarithmic growth phase (typically ~ 25–80% confluence) to plate cells for subsequent drop tower experiments. For each drop tower culture set up, 50,000 or 100,000 fibroblasts are plated in 1 mL of complete media. Each cell-culture is then monitored in real-time while incubating with a 10× phase objective and 25 regions of interest (ROI) at a frequency of every 30 min (Incucyte, Essen Bioscience). Confluency of each individual culture is calculated via automated segmentation of each ROI utilizing an image recognition algorithm trained on Hs27 cells offered through the Incucyte software (see the Supplementary Movie 6 (5pfs), Essen BioScience, INC., Ann Arbor). The resulting growth-curve of a single culture set up is the average of 25 individual ROI confluence measurements. Typically, eight cell culture setups are prepared and monitored in parallel. After the cultures reach the approximate middle of the logarithmic growth phase (approximately 35–40% confluence), each cell culture setup is assembled with the holder for drop tower experiments (Step-ii in Fig. 1b). Each drop tower test takes approximately 15 min and is conducted under aseptic conditions, minimizing deviations of in-vitro conditions. For each batch of experiments, one culture set up remains in the incubator as a reference, while another undergoes the same steps and conditions of a drop tower test without any impact forces being distributed on the culture as a control. The remaining cultures are utilized for drop tower tests.

### Drop tower experiments

To simulate realistic mechanical impact on biological systems, a recently developed system, called the integrated drop tower system^21^, is utilized. The integrated drop tower system (Fig. 1a) consists of a conventional drop tower impact system (Dynatup 9210, Instron, Norwood, MA), two high speed cameras (Fastcam SA-X2RV, Photron, San Diego, CA), a cell culture setup (see the SI), A three-axis ICP-based accelerometer (model #356A01, PCB Piezotronics, Depew, NY), and a data acquisition system (National Instruments Corp., TX). One or two independent high speed cameras are mounted on two camera stands such that the cameras can concurrently capture the front or/and side views of the cuvette, typically at 50 k frames per second (fps). The plastic sample holder is manufactured using a MakerBot Replicator 2 (MakerBot Industries, LLC, Brooklyn, NY) 3D printer using MakerBot PLA (polylactic acid) plastic filament. Printing is done using default settings, with the exception of the infill being set to 100%. The accelerometer is glued onto the holders using 5-min epoxy. We use *soft form 1* (1 mm thick, 50A silicone rubber, McMaster-Carr, Princeton, NJ) and *2* (12 mm thick, silicone polymer closed cell sponge #7904, MTI Groendyk Inc., Richmond, VA) to control the characteristics of impact. Data acquisition is performed using a system consisting of an NI PXIe-8135 embedded controller and an NI PXI-6115 multifunction I/O module using SignalExpress 2014 data acquisition software (all from National Instruments Corp., Austin, TX). The accelerometer (model #356A01, PCB Piezotronics, Depew, NY) is connected to three channels of the data acquisition system through an ICP signal conditioner (model #480B21, PCB Piezotronics). Data are acquired at a rate of 1 MHz and triggered off the vertical axis of the accelerometer. A pre-trigger buffer is used to capture the entirety of the signal event. Upon triggering the data acquisition system is programed through SignalExpress to output a TTL pulse to the two Photron cameras which are set up with a pre-trigger buffer equal in time to the data acquisition system’s buffer. The time delay between the triggering of the data acquisition system and output of the TTL is less than a single sample (< 1 µs). To reliably measure pressure, a PCB 113B21 pressure sensor was used (PCB Piezotronics, Depew, NY). This has a 2,758 kPa max range, with a rise time of less than 1 µs.

### PI

To access the plasma membrane integrity, propidium iodide (PI, ~ 700 Da, abcam ab14083, Cambridge, UK) is used. During the assembly of the cell culture setup (Fig. 1b), PI solution diluted in cell culture media is added into a petri dish to achieve the final PI concentration of 10 µg/ml. After completion of a drop tower experiment, each petri dish is washed with PBS and then regular cell culture media is added for live cell imaging of the fibroblasts during cell culture.

## Supplementary information

Supplementary Movie 1.

Supplementary Movie 2.

Supplementary Movie 3.

Supplementary Movie 4.

Supplementary Movie 5.

Supplementary Movie 6.

Supplementary Figures.
