# Supplementary material for: Mechanisms of cell damage due to mechanical impact: an in vitro investigation
Source: Sci Rep. 2020 Jul 20;10:12009. doi: 10.1038/s41598-020-68655-2 (PMC7371734; doi:10.1038/s41598-020-68655-2)
Supplement: Supplementary file 7 — Supplementary Figures. [file 41598_2020_68655_MOESM7_ESM.docx]

**Mechanisms of Cell Damage due to Mechanical Impact – An *In Vitro* Investigation**

Wonmo Kang^1*^, Michael Robitaille^2^, Marriner Merrill^2^, Kirubel Teferra^2^, Chunghwan Kim^1^, and Marc P. Raphael^2┼^

^1^Arizona State University, Tempe, AZ 85287

^2^US Naval Research Laboratory, Washington, DC 20375

*Email: [wonmo.kang@asu.edu](mailto:wonmo.kang@asu.edu)

^┼^Email: [marc.raphael@nrl.navy.mil](mailto:marc.raphael@nrl.navy.mil)

**Supplement**

1. Cell culture setup for drop tower experiments


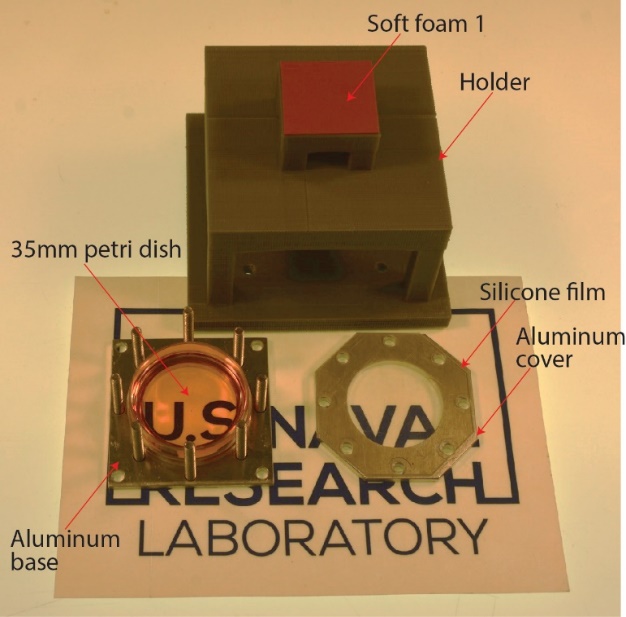


Figure S1 An optical image of a cell culture setup for a drop-tower experiment.

1. Control of impact characteristics using soft forms


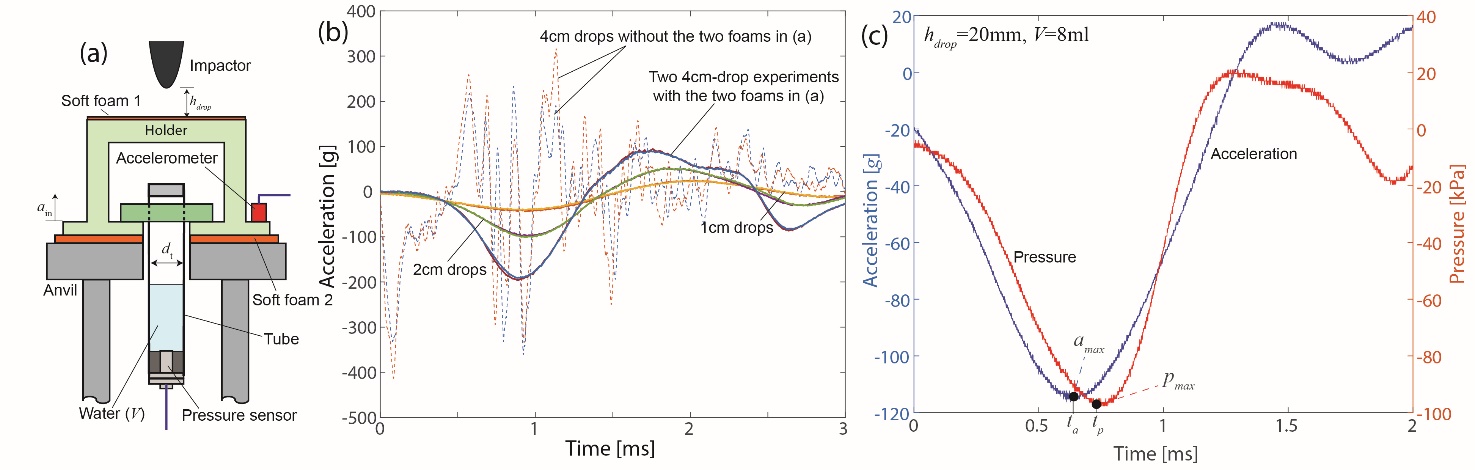


Figure S2 (a) Schematic of the experimental setup, (b) Acceleration response of the holder with and without use of soft foams and (c) the experimental comparison of acceleration and pressure with use of the foams during impact. The significant difference is observed in the measured acceleration signals in (b) when compared the two cases, i.e., with and without the soft foams. Compared to the latter (dotted lines in b), the former (solid lines in b) has much smoother acceleration profiles with much longer time scale (~1ms).

1. The Navier-Stokes equations for incompressible Newtonian fluid in the cylindrical polar coordinates

Consider the schematic that presents the cell culture setup with an emphasis on time-dependent pressure profile in the cell culture media during impact where $d$ and $H$ in Figure S1a are the diameter and height of the cell culture chamber, respectively. $r$, $\theta$, and $z$ in Figure S1b are the radial, angular, and vertical coordinates with respect to the origin at the center of the bottom surface of the cell culture chamber. Upon mechanical impact on the holder, the cell culture setup is rapidly accelerated by$a_{in}$ that results in acceleration-induced pressure ($p_{a}$) in the cell culture media where $a_{in}$ and $p_{a}$ are functions of time ($t$).


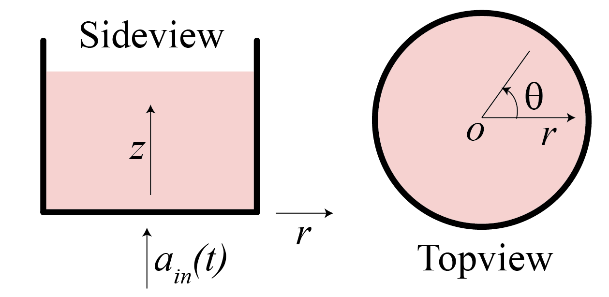


Figure S3 A schematic for the liquid in a cylindrical container with an opening at the top. The container is accelerated by $a_{in}$ during impact. $r, \theta,$ and $z$ establish the cylindrical polar coordinate system.

The governing equation of motion for incompressible Newtonian fluid (the Navier-Stokes equations) in the container can be written as

In $r$-direction:

$\rho\left( \frac{\partial v_{r}}{\partial t}+v_{r}\frac{\partial v_{r}}{\partial r}+\frac{v_{\theta}}{r}\frac{\partial v_{r}}{\partial\theta}-\frac{v_{\theta}^{2}}{r}+v_{z}\frac{\partial v_{r}}{\partial z} \right)=-\frac{\partial p}{\partial r}+\rho G_{r}+\mu[\frac{1}{r}\frac{\partial}{\partial r}\left( r\frac{\partial v_{r}}{\partial r} \right)-\frac{v_{r}}{r^{2}}+\frac{1}{r^{2}}\frac{\partial^{2}v_{r}}{\partial\theta^{2}}-\frac{2}{r^{2}}\frac{\partial v_{\theta}}{\partial\theta}+\frac{\partial^{2}v_{r}}{\partial z^{2}}]$ Eq. S1

In $\theta$-direction:

$\rho\left( \frac{\partial v_{\theta}}{\partial t}+v_{r}\frac{\partial v_{\theta}}{\partial r}+\frac{v_{\theta}}{r}\frac{\partial v_{\theta}}{\partial\theta}+\frac{v_{r}v_{\theta}}{r}+v_{z}\frac{\partial v_{\theta}}{\partial z} \right)=-\frac{1}{r}\frac{\partial p}{\partial\theta}+\rho G_{\theta}+\mu[\frac{1}{r}\frac{\partial}{\partial r}\left( r\frac{\partial v_{\theta}}{\partial r} \right)-\frac{v_{\theta}}{r^{2}}+\frac{1}{r^{2}}\frac{\partial^{2}v_{\theta}}{\partial\theta^{2}}+\frac{2}{r^{2}}\frac{\partial v_{r}}{\partial\theta}+\frac{\partial^{2}v_{\theta}}{\partial z^{2}}]$ Eq. S2

In z-direction:

$\rho\left( \frac{\partial v_{z}}{\partial t}+v_{r}\frac{\partial v_{z}}{\partial r}+\frac{v_{\theta}}{r}\frac{\partial v_{z}}{\partial\theta}+\frac{v_{r}v_{\theta}}{r}+v_{z}\frac{\partial v_{z}}{\partial z} \right)=-\frac{\partial p}{\partial z}+\rho G_{z}+\mu[\frac{1}{r}\frac{\partial}{\partial r}\left( r\frac{\partial v_{z}}{\partial r} \right)+\frac{1}{r^{2}}\frac{\partial^{2}v_{z}}{\partial\theta^{2}}+\frac{\partial^{2}v_{z}}{\partial z^{2}}]$ Eq. S3

where $\rho, \mu$ and $p$ are density, viscosity, and pressure of fluid. $v_{r},v_{\theta},v_{z}$ and $G_{r},G_{\theta},G_{z}$ are velocity of fluid and the acceleration of gravity in $r, \theta,z$-directions, respectively.

Due to axial symmetry about $z$-axis, the Navier-Stokes equations become independent of $\theta$and, as a result, all terms with a partial derivative of $\theta$ in Eq. S1-S3 are zero ($\partial/\partial\theta=0$). In addition, all external forces applied to the cell culture media through the solid-liquid interfaces including $\left( r,z \right)=(d/2, z)$ and $=(r, 0)$ are in $z$-direction, which indicates that flow of the cell culture media in $r$- and $\theta$-directions is zero ($v_{r}=v_{\theta}=0$). Now to satisfy incompressibility, we must have$\partial v_{z}/\partial z=0$. Using the assumptions above, Eq. S1-S3 become

$$\left\{ \begin{aligned} r-direction: \frac{\partial p}{\partial r}=0 \\ \theta-direction: \frac{\partial p}{\partial\theta}=0 \\ z-direction: \rho\left( a_{in}-g \right)=-\frac{\partial p}{\partial z} \end{aligned} \right.$$

We experimentally validate that the assumptions on$v_{r}, v_{\theta}$, and $\partial v_{z}/\partial z$ above are reasonable. For this, we have prepared a pure water sample in a transparent cuvette and monitored the meniscus profile (see the highlighted areas by red ellipses in Fig. S2) near the top of the cuvette using a high-speed camera movie during impact. Note that the two high-speed camera images are taken $t=0$ and $=0.42$ ms, which correspond to the onset of impact and the time point when amplitude of impact-induced acceleration is maximum. Because any non-zero flow velocity of the water in $r$- or $z$-direction would cause changes in the meniscus profile, we have concluded that our assumptions is reasonable during initial acceleration.


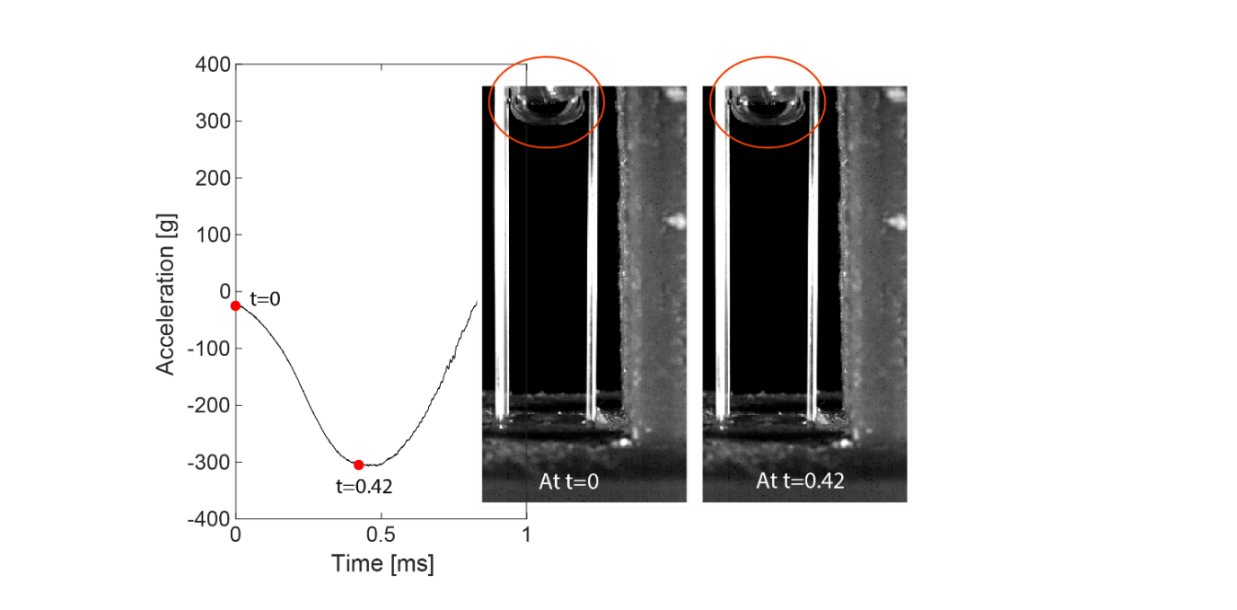


Figure S4 The shape of water maniscus during impact at *t*=0 and =0.42ms

1. Heterogeneity in spatial distribution of cell population

Average of each ROI with standard errors (95% interval). Here our focus is to provide detailed data for Figure 4-c consisting of 6 independent petri dishes. As a reminder, relatively lower drop heights (from 5cm to 20cm shown in Figure 4a-b) do not result in measurable chance in local as well as average


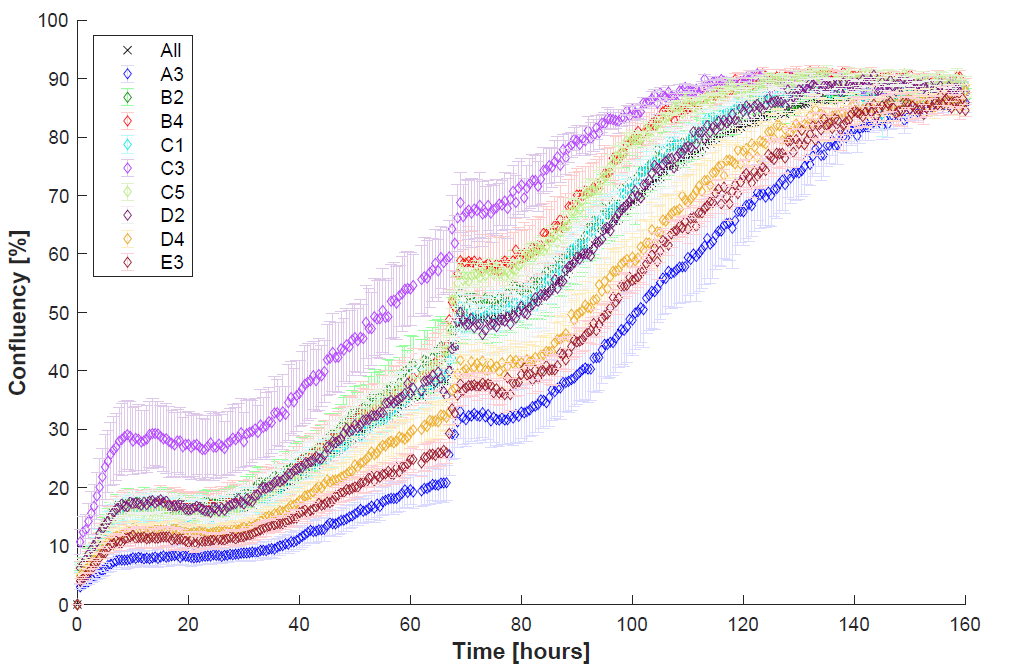

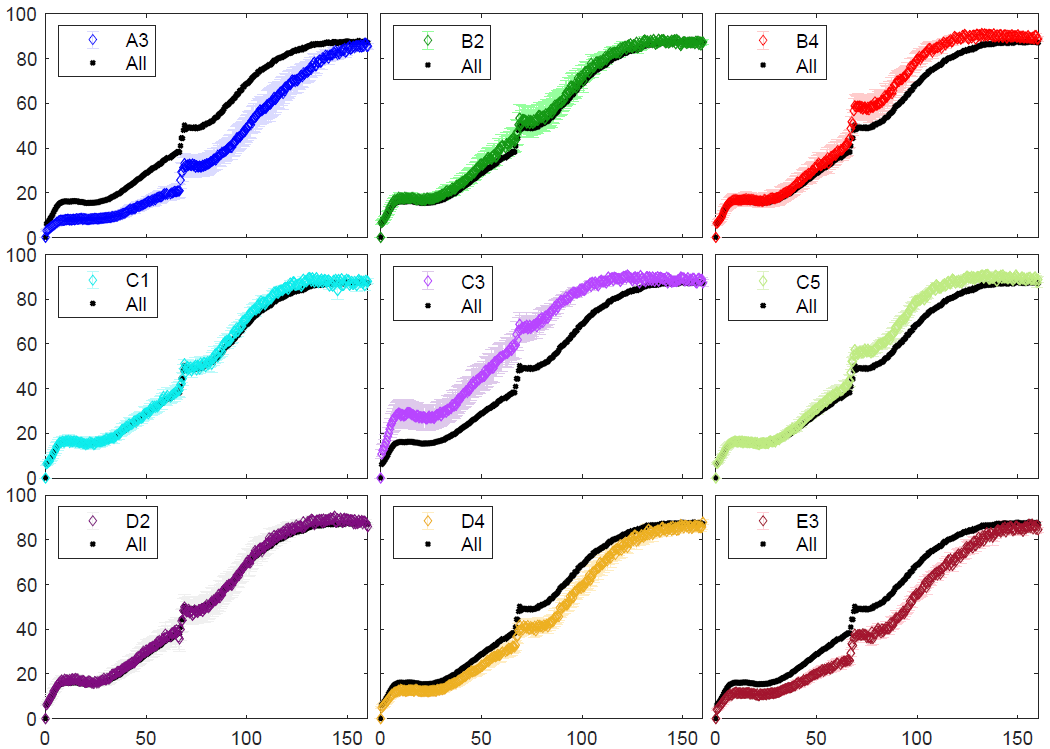


Figure S5-1 Local and average confluency curves for Control 1 from Passage 11. The upper panel shows the confluency curves at each ROI with the standard error (95% interval) and the average confluency curve (designated by “all” in legend) from the nine ROIs. The lower panel shows detail from each ROI where the local confluency curve is compared with the average confluency curve (see the legend in each plot). The horizonal and vertical axes are Time [hours] and Confluency [%], respectively.


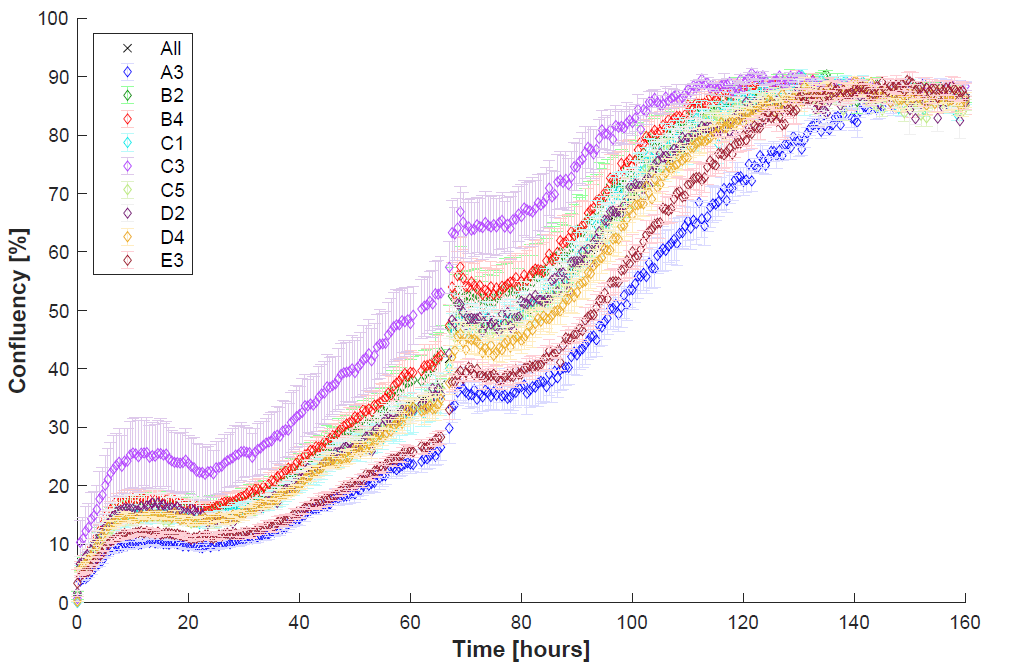


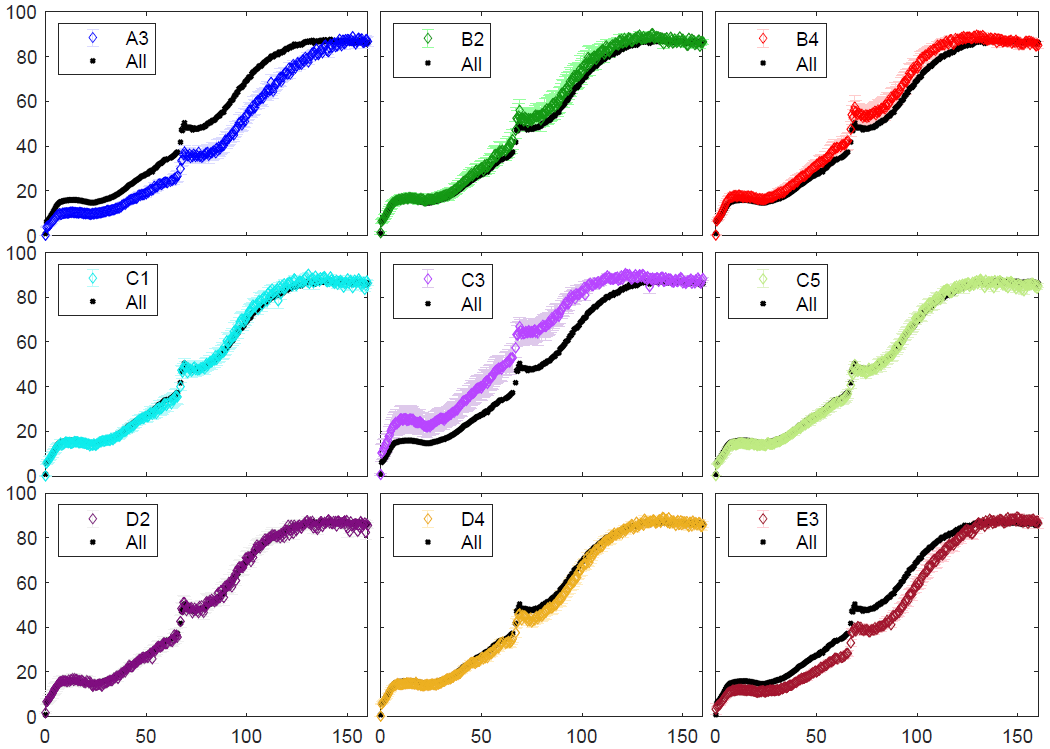


Figure S5-2 Local and average confluency curves with the standard error (95% interval, error bars are shown each plot) for Control 2 from Passage 11 (the average confluency curves are shown in Figure 4d). The upper panel shows the confluency curves at each ROI and the average confluency curve (designated by “all” in legend) for the nine ROIs. The lower panel shows details for each ROI with the standard error where the local confluency curve is compared with the average confluency curve (see the legend in each plot). The horizonal and vertical axes are Time [hours] and Confluency [%], respectively.


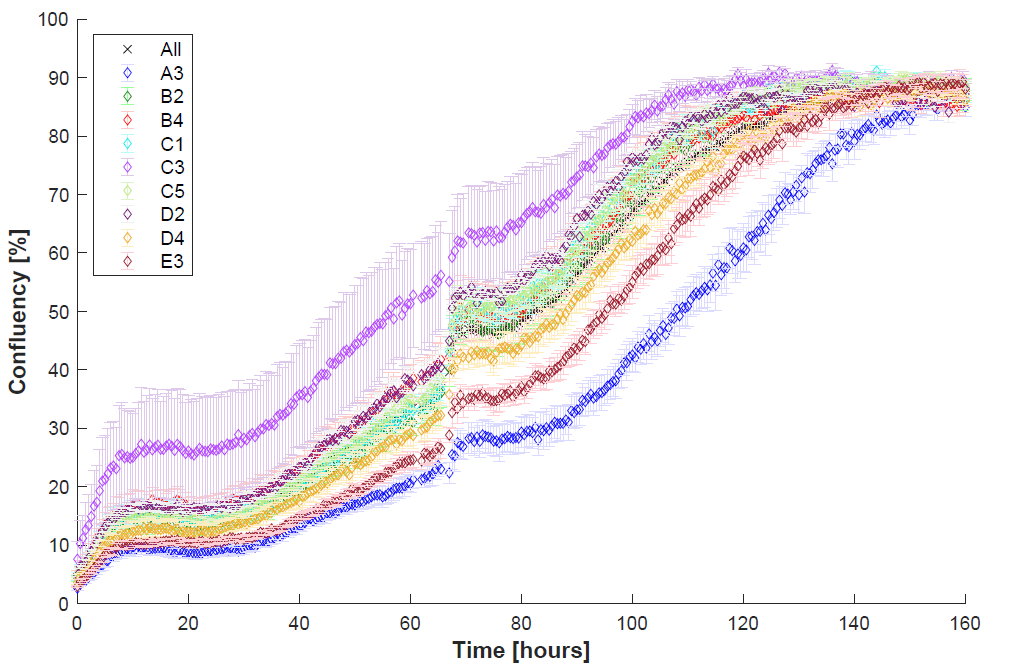


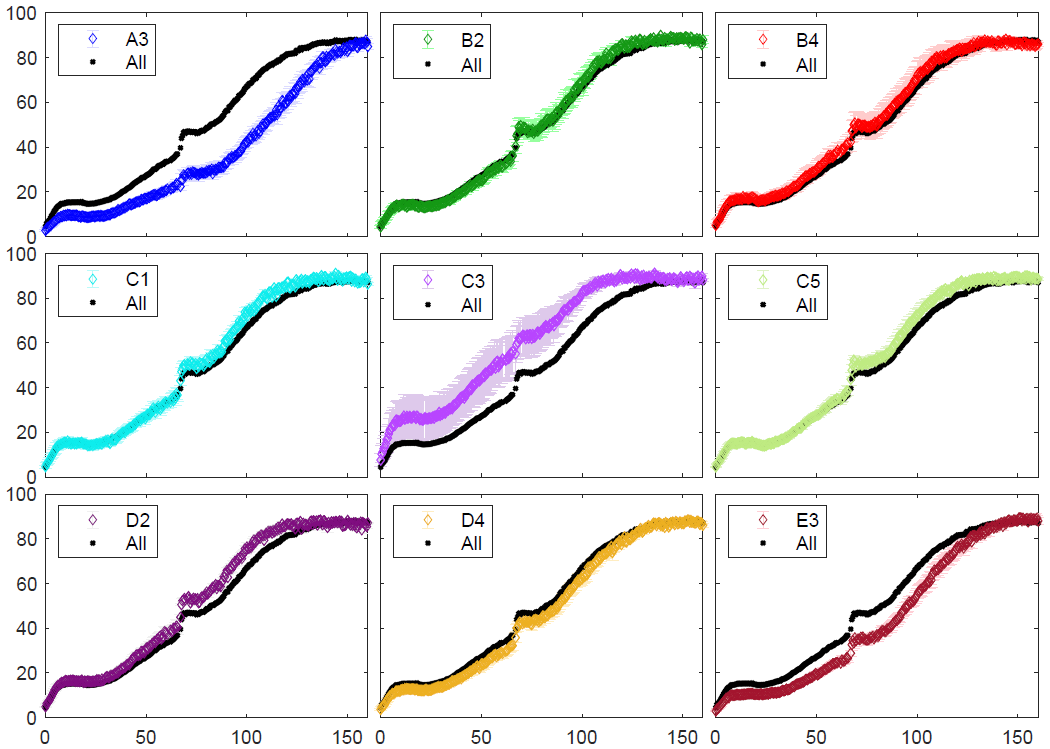


Figure S5-3 Local and average confluency curves with the standard error (95% interval, error bars are shown each plot) for 30cm drop 1 from Passage 11 (the average confluency curves are shown in Figure 4d). The upper panel shows the confluency curves at each ROI and the average confluency curve (designated by “all” in legend) for the nine ROIs. The lower panel shows details for each ROI with the standard error where the local confluency curve is compared with the average confluency curve (see the legend in each plot). The horizonal and vertical axes are Time [hours] and Confluency [%], respectively.


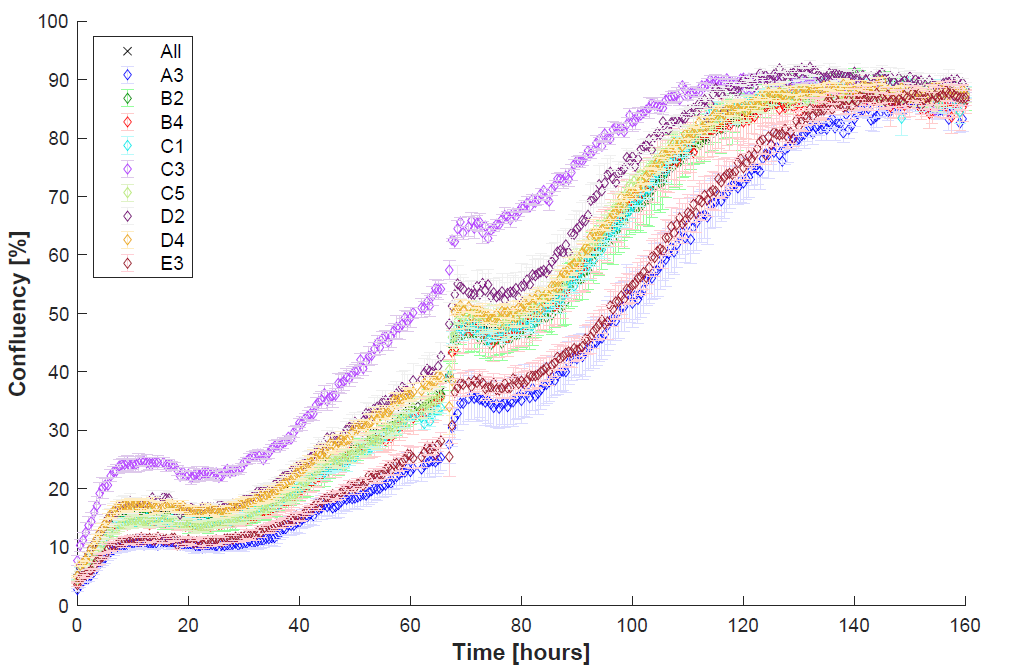


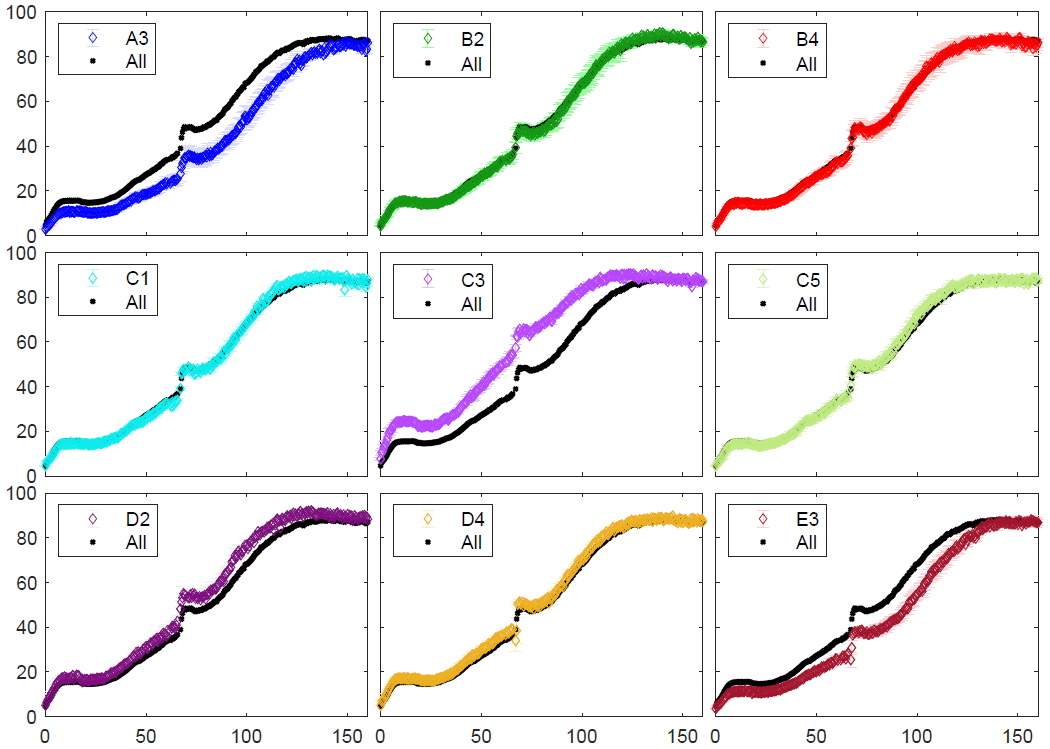


Figure S5-4 Local and average confluency curves with the standard error (95% interval, error bars are shown each plot) for 30cm drop from Passage 11 (the average confluency curves are shown in Figure 4d). The upper panel shows the confluency curves at each ROI and the average confluency curve (designated by “all” in legend) for the nine ROIs. The lower panel shows details for each ROI with the standard error where the local confluency curve is compared with the average confluency curve (see the legend in each plot). The horizonal and vertical axes are Time [hours] and Confluency [%], respectively.


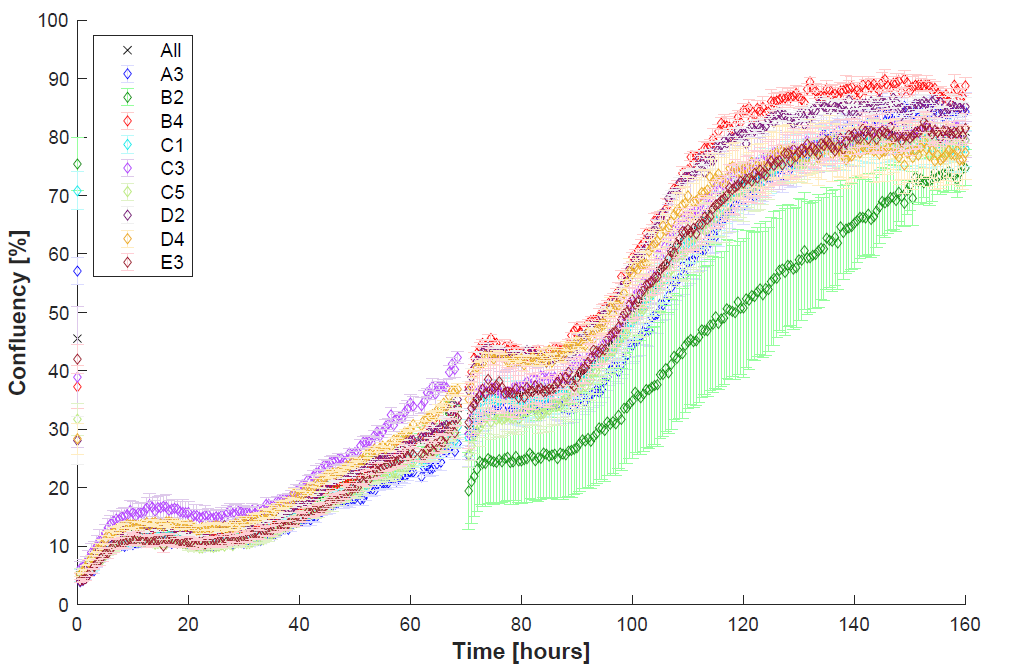


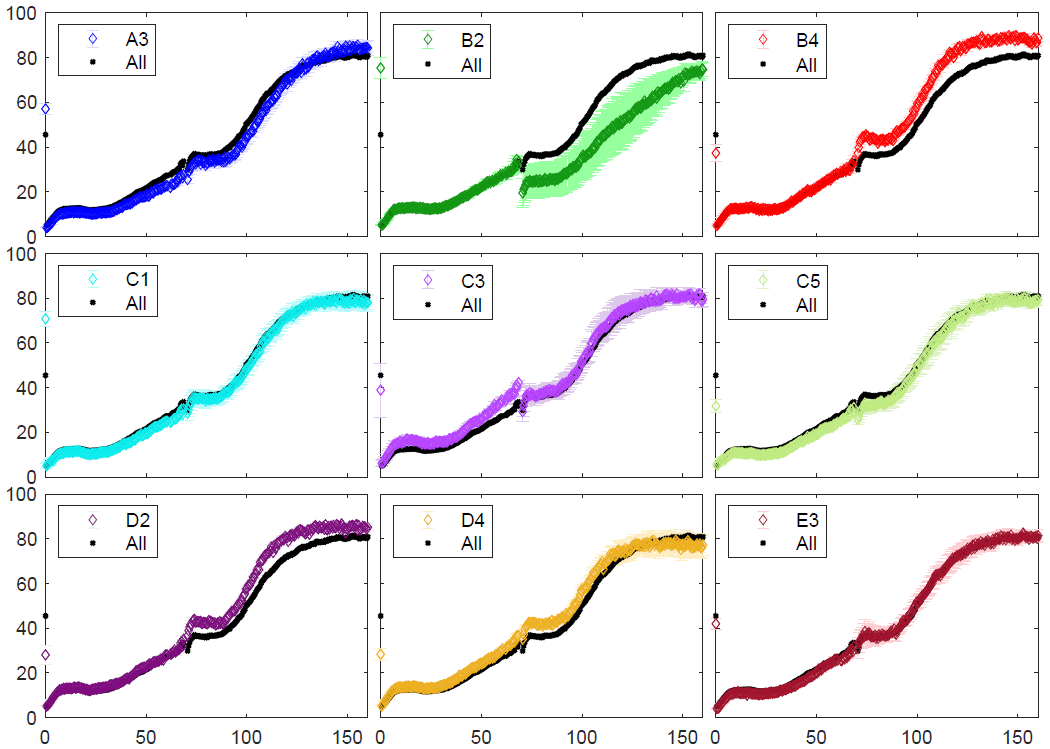


Figure S5-5 Local and average confluency curves with the standard error (95% interval, error bars are shown each plot) for 45cm drop 1 from Passage 11 (the average confluency curves are shown in Figure 4d). The upper panel shows the confluency curves at each ROI and the average confluency curve (designated by “all” in legend) for the nine ROIs. The lower panel shows details for each ROI with the standard error where the local confluency curve is compared with the average confluency curve (see the legend in each plot). The horizonal and vertical axes are Time [hours] and Confluency [%], respectively.


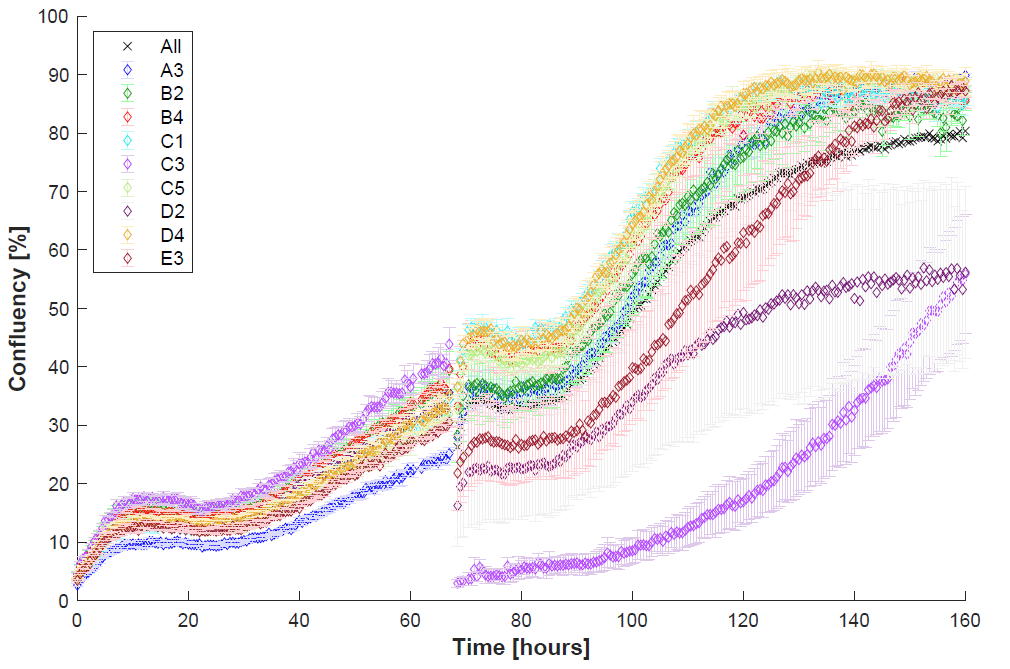


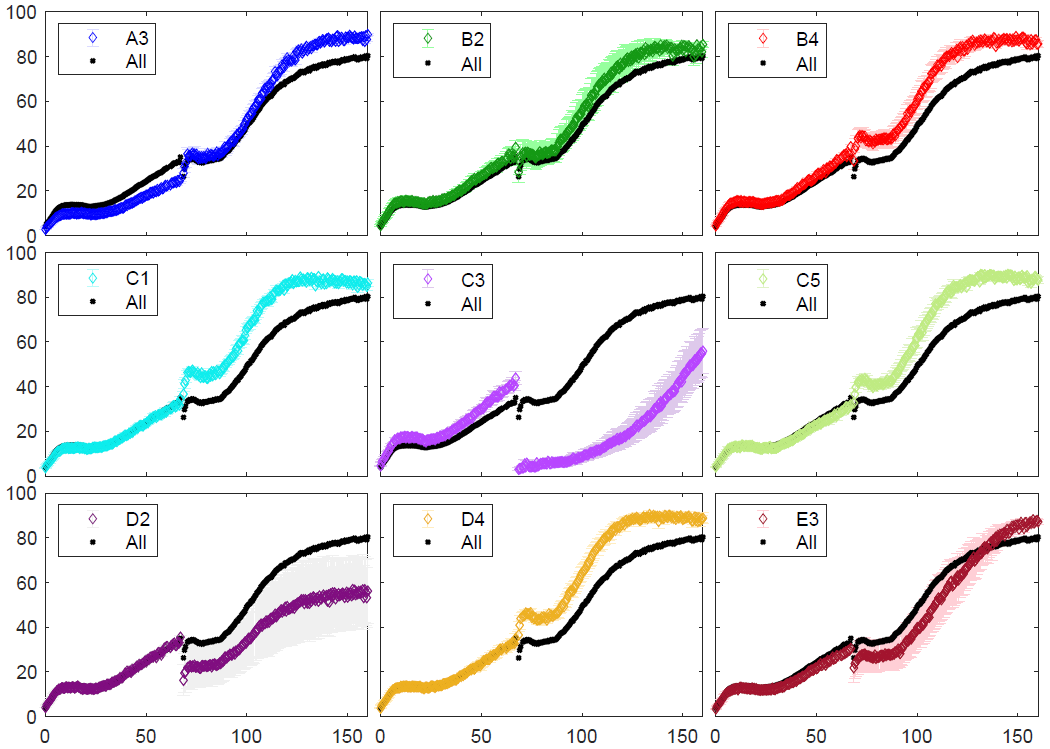


Figure S5-6 Local and average confluency curves with the standard error (95% interval, error bars are shown each plot) for 40cm drop 2 from Passage 11 (the average confluency curves are shown in Figure 4d). The upper panel shows the confluency curves at each ROI and the average confluency curve (designated by “all” in legend) for the nine ROIs. The lower panel shows details for each ROI with the standard error where the local confluency curve is compared with the average confluency curve (see the legend in each plot). The horizonal and vertical axes are Time [hours] and Confluency [%], respectively.

1. Localized damage due to cavitation

Our criterion for the damaged area is a detectable decrease with a statistical significance in local confluency by directly comparing live cell images right before and after impact. As an example, each area in ROI: B4 and C3 has very different confluency curves (see Figure S6-1). The confluency curves for the four neighboring areas (*A*1-4) within ROI: B4 have a very similar trend and are comparable to the confluency curve for the control which was not subjected to any impact (see Figure S6-1b and Figure 4c, control 2). We utilized our unique capability to monitor live cells in real time to quantify a sudden decrease in the cell confluency that is specific to local areas. Note that our image analysis for ROI: C3 (see Figure S6-2) clearly shows a significant change in the cell confluency where the red region is the area of cells determined by the cell image analysis software.


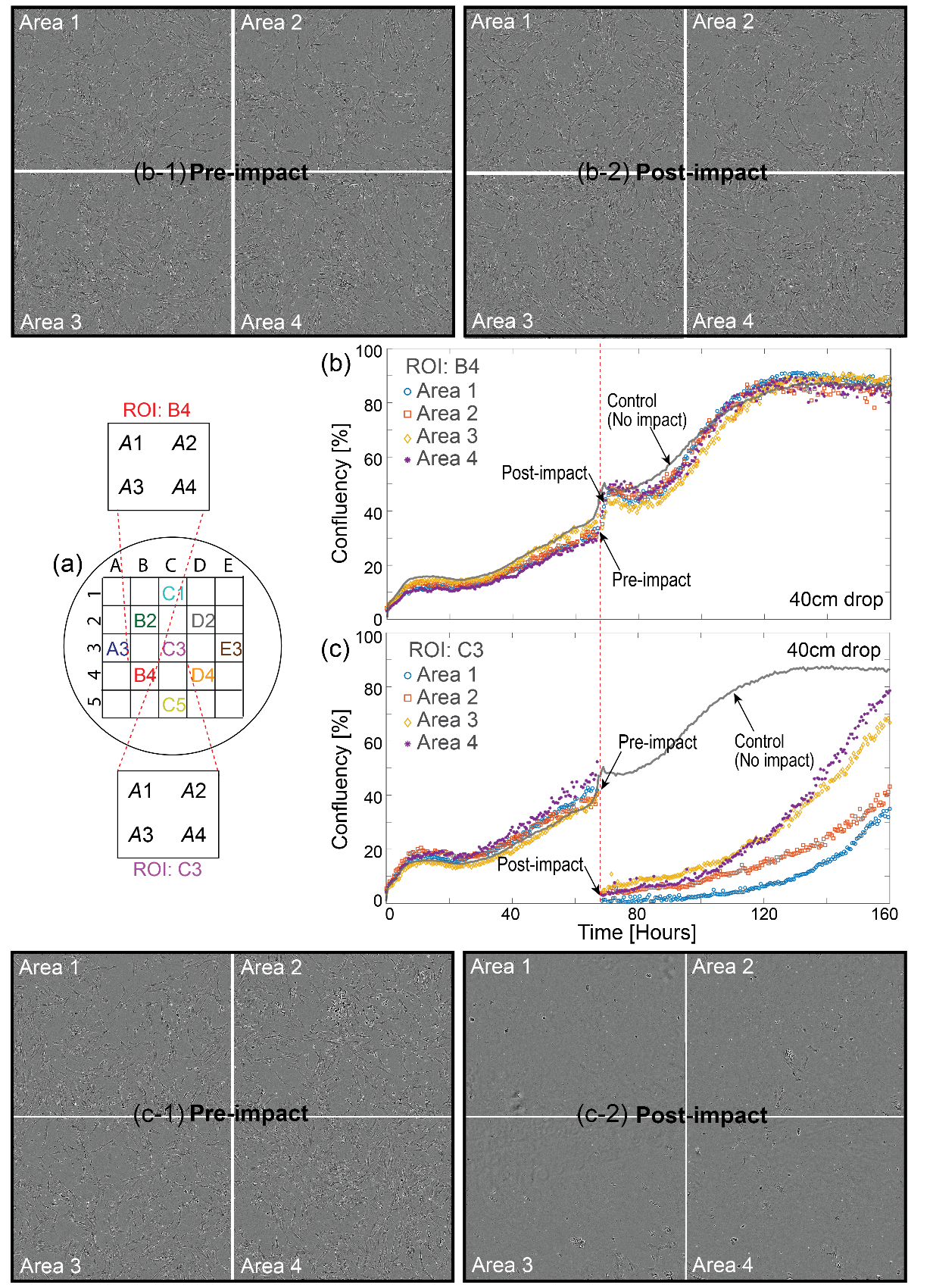


Figure S6-1 Identification of damaged areas due to applied impact. (a) Region of interests on a petri dish. Each ROI consists of four areas (*A*1, *A*2, *A*3, and *A*4). To determine damaged areas, the image analysis algorithm discussed in the method section is used to monitor confluency in each area. (b) and (c) show confluency curves in B4 and C3, respectively. Note that confluency curves in b and c have very different trend after impact. (b-1 and -2) and (c-1 and -2) show the live cell images just before and after impact in B4 and C3, respectively.


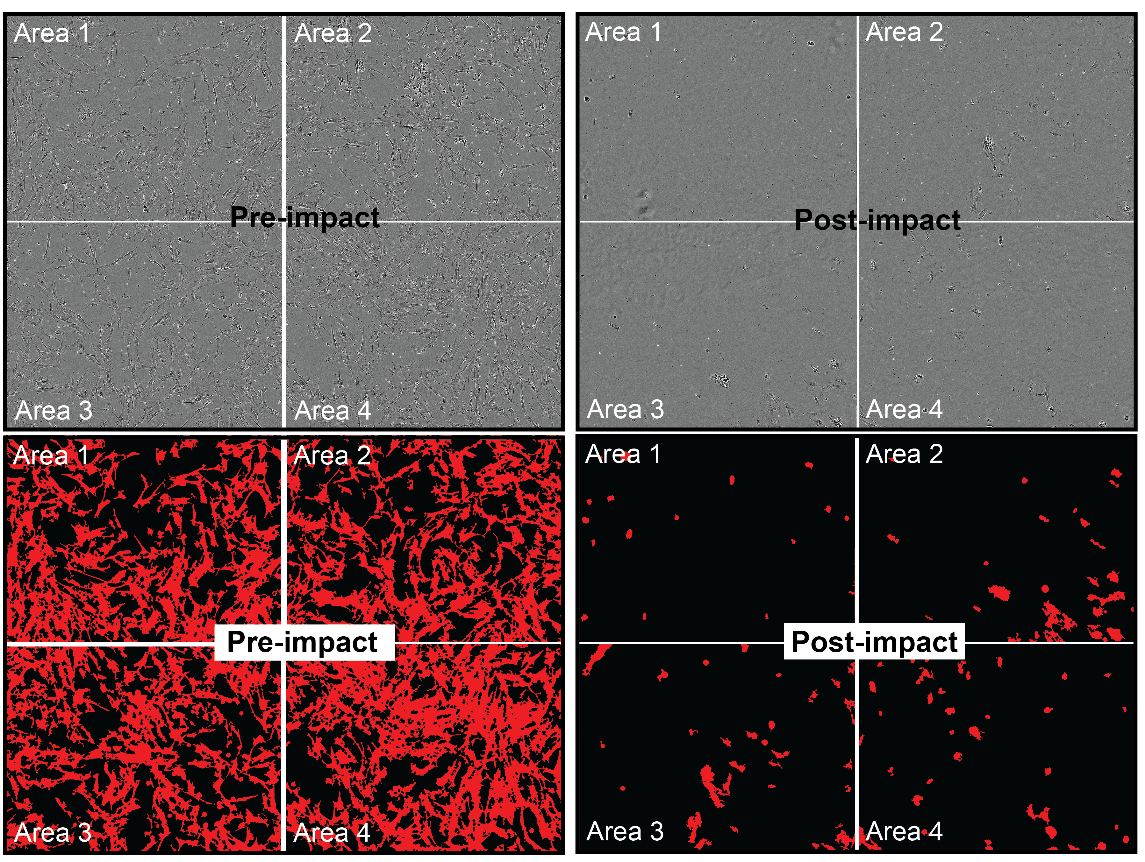


Figure S6-2 Upper panel: live cell images Figure S6c-1 and -2. Lower panel: the corresponding area of cells (red areas) recognized by software.


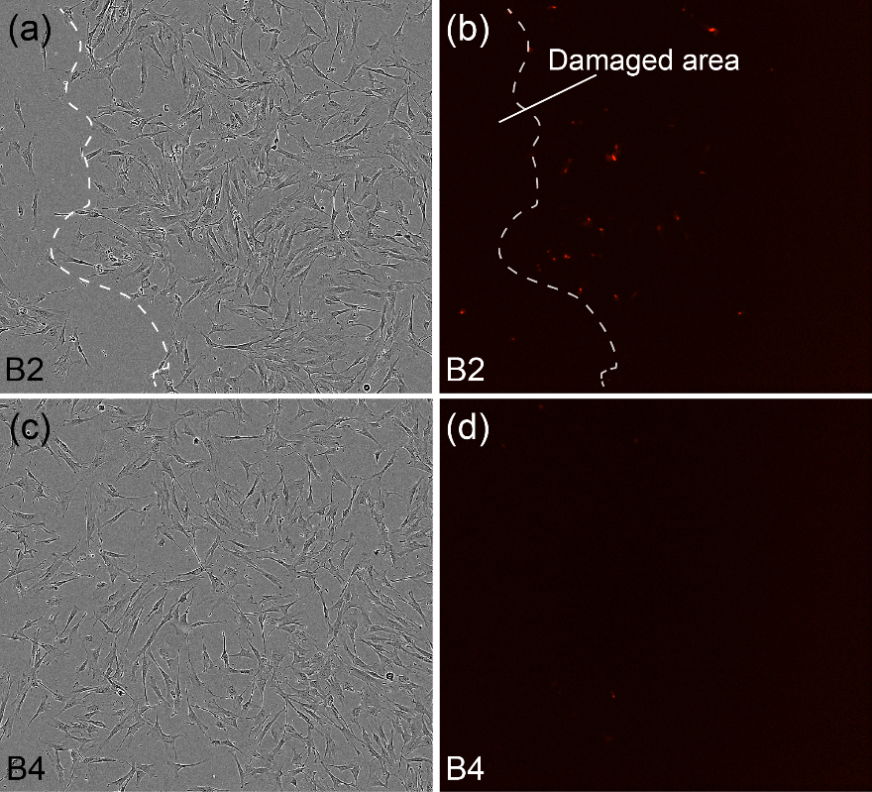


Figure S6-3 Phase and red fluorescence images after 40cm-drop impact: (a)-(b) near the damaged area (B2) by cavitation and (c)-(d) a few millimeters away from B2 (see Figure 3 and 4).
